# Do Social Pensions Affect the Physical and Mental Health of Rural Children in China? An Intergenerational Care Perspective

**DOI:** 10.3390/ijerph19073949

**Published:** 2022-03-26

**Authors:** Sipei Xu, Jia Zhang

**Affiliations:** 1 School of Public Affairs, Zhejiang University, Hangzhou 310000, China; 22122170@zju.edu.cn; 2 College of Education, Zhejiang University, Hangzhou 310000, China

**Keywords:** social pensions, New Rural Pension Scheme in China, intergenerational care, rural children’s mental health, rural children’s physical health

## Abstract

Research Purpose: This study aimed to explore the effect of China’s New Rural Pension (NRP) on the physical and mental health of rural children from the perspective of intergenerational care, and to examine whether family childcare types and the child’s gender affect the relationships between social pensions and the physical and mental health of rural children. Methods: We used data from the 2016 China Family Panel Studies (CFPS) of the China Social Science Survey Center, a nationally representative sample at the individual, family, and county levels from 25 provinces (cities and districts) in China. A total of 2142 sets of valid samples of children, the elderly, family economic and social conditions, and basic family information were retained after data screening. The regression discontinuity (RD) method was employed for the statistical analyses. Results: The NRP had a significant effect on both the mental health (β = −2.818, *p* < 0.1) and physical health (β = −2.214, *p* < 0.1) of rural children. This effect varied with the family childcare type and child’s gender. Conclusions: We reveal a positive effect of the NRP on the physical and mental health of rural children. Therefore, the establishment of a social pension system may be used as an effective approach to enhance the health of rural children. The impact of the NRP on the physical and mental health of children differs with the family childcare type and their gender, which should be taken into consideration when using social pensions to enhance child health.

## 1. Introduction

Social pensions, as a vital part of national welfare, can guarantee the basic living needs of people (especially vulnerable groups), narrow the income gap, and smooth economic fluctuations, thus contributing to social stability [1]. The development and influence of social pension systems have been widely discussed around the world. Studies have examined how to develop a social pension system to adapt to the economic development and social needs, such as the communication mechanism between the government and the public in the process of pension decision making [2], the design of personal income distribution [3], the relationship between social pensions and the political economy [4], and the pension system’s unification [5], sustainability [6], and application [7], among other aspects.

An increasing number of studies have explored the effects of social pensions on the physical and mental health of those receiving them [8,9,10,11], and differences between developed and developing countries have been detected. In developed countries, such as European societies, social pensions have a double-edged effect; that is, they help to reduce old-age poverty, but increase social inequalities [12]. In contrast, in developing countries, such as Vietnam and India, social pensions can improve the economic income of poor elderly people, while also narrowing the income gap among them [13,14]. Furthermore, social pensions also can exert a positive influence on labor supply [15]. The impacts of social pensions on other family members, such as transfer payments between the elderly and their adult children [16], have also been investigated. However, research on the effects of social pensions on the health of grandchildren remains limited. Although recent studies [17,18] have begun to pay attention to the relationship between social pensions and elderly’s grandchildren’s physical health, the findings of these studies need further verification. Moreover, the effects of social pensions on elderly’s grandchildren’s mental health have been largely ignored, thus calling for more investigation.

The physical and mental health of children, as significant predictors of their human capital development, have received considerable attention around the world [19,20]. Children with poor health will probably suffer from a low level of labor income, social status, and creativity in adulthood [21], and may be drawn into the “nutrition-poverty” trap. This means that the lack of nutrition in childhood severely affects people’s human capital development in a poor family, leading to a vicious cycle in which their children are also undernourished due to poverty [22]. At present, the child health problem is particularly prevalent in developing countries [23]. Research (see, e.g., [24]) has shown that family care is vitally important for children, and both the physical and mental health of children in their early stages of development are closely related to family care [25]. Although intergenerational care constitutes an important form of family care [26], as the main provider of intergenerational care, the life quality of the elderly and its influence on the physical and mental health of children need to be researched in more detail. Specifically, studies have shown that the income level of the elderly produces a significant and positive effect on the physical health of children [27], and their care style (specifically, the behavior of spoiling children) can be detrimental to children’s mental health [28,29]. Social pensions are closely related to the income level [30,31] and the care style (i.e., full-time care or part-time care) [32,33] of the elderly. Therefore, it is important to examine the effect of social pensions on children’s physical and mental health, in order to enhance our knowledge of the effect of social pensions from the intergenerational care perspective.

In China, a social pension system (i.e., the New Rural Pension Scheme) was developed for rural residents in 2009. Although studies have explored the impact of the New Rural Pension (NRP) on the elderly and the transfer payment between elderly and their adult children, only a few studies have been conducted to explore the effect of the NRP on the physical health of elderly’s grandchildren [34,35], and the effect of the NRP on the mental health of elderly’s grandchildren is still under-explored. Considering that intergenerational care is very common [36] in rural China, it is important to examine the effect of the NRP on the physical and mental health of children, and thus increase our understanding of how social pensions affect rural families.

The purpose of this study was to explore the effect of social pensions on the physical and mental health of children, and to contribute to the existing literature in the following ways: First, as existing studies have mainly focused on the impact of social pensions on receivers and their adult children, we further explore the spillover effect of social pensions between elderly and their grandchildren, which can help in evaluating the effect of social pensions from the perspective of intergenerational childcare within the family. Second, considering that research on the effect of social pensions on the health of children is limited, we explore the impact of social pensions on both the physical and mental health of children. Third, while existing literature suggests that different family childcare types and children’s gender can result in different external environments and child growth conditions, which are closely related to children’s physical and mental health, we further explore the heterogeneity effect of social pensions on these two factors in the relationship between social pensions on the health of children. This can provide a new perspective for policy research and evaluation regarding the effect of social pensions on child development. Finally, we adopt a fuzzy regression discontinuity strategy to better address the endogenous problem caused by the different behaviors of social pension recipients and non-recipients, which can provide further evidence for using regression discontinuity to evaluate the effect of social pensions.

The remainder of this paper is structured as follows: Section 2 provides the theoretical foundation. Section 3 illustrates the New Rural Pension Scheme in China. Section 4 describes the methodology. Section 5 outlines our empirical results. Section 6 provides a discussion of the results. Finally, Section 7 summarizes our conclusions.

## 2. Theoretical Foundation

### 2.1. The Impact of Elderly’s NRP Reception on Children’s Health Development

We present the theoretical inferences to illustrate how the NRP has an impact on the health of rural children from the perspective of intergenerational care.

#### 2.1.1. The Income Effect and Substitution Effect of the NRP on the Elderly

Intergenerational exchange theory originates from social exchange theory, which emphasizes the “loan-repayment contract” and takes reciprocal equilibrium as the driving force to sustain social exchange [37]. Within families, parents and children reach a reciprocal equilibrium between giving and repaying, due to economic interests, moral obligations, and emotional needs. Offspring support is a reciprocal exchange behavior of children, based on their parent’s child-raising and support in early life [38]. In other words, due to economic and social rationality, when their children go out to work, rural elderly people take on the responsibility of caring for grandchildren, through which they hint that their children should take on the responsibility of taking care of them in the future. When the NRP raises the welfare level of the insured elderly, it naturally affects the level of intergenerational care, which further produces an impact on the human capital accumulation (e.g., physical and mental health) of rural children.

Specifically, the impact of the NRP on the elderly is reflected in two aspects: the income effect and the substitution effect. The income effect refers to the NRP increasing the disposable income of the elderly and raising their consumption level. This can further increase the consumption of the elderly allocated to children in a fixed proportion [35]; improve the household food security and dietary quality, which are beneficial to child nutritional intake [39]; upgrade household facilities, which may have positive health consequences [40]; and relieve the stress of the elderly (family childcare providers) related to poverty, by improving the household economic status. This may, in turn, result in more positive childcare behavior; that is, the elderly may have better health consciousness regarding children and stronger economic capability to provide children with health services [41].

The substitution effect is both direct and indirect. The direct effect refers to the NRP directly increasing the time spent by insured elderly people in caring for children; that is, the NRP increases the wealth level of insured elderly people and relaxes their financial constraints [42], thus prompting rural elderly people to take the initiative to increase the frequency and intensity of childcare, which further improves the physical and mental health of the children. The indirect effect refers to the NRP helping to reduce the labor burden of rural elderly people and increase their daily leisure time [43], which may increase the time that rural elderly people spend on caring for their grandchildren, thus helping improve the physical and mental health of rural children.

#### 2.1.2. Children’s Health Investment Decision Making

According to Grossman [44] and Yu et al. [35], we established the following child health investment model:

(1) $$U_{c}=U\left(Z,H\right),$$

(2) TC+TH+TL=Ω,

where Equation (1) represents the utility function of children; $U_{c}$ indicates the utility of children; Z represents the consumption of children other than medical treatment; and H represents the health time of children (i.e., the time when children are in a state of physical and mental health). Equation (2) represents the time constraint of children, in which TC, TH, and TL represent the time children spend on consumption, the time they spend on improving their health, and the time they are not in good physical and mental state, respectively, where TC + TH = h, and Ω indicates the total time of children. Furthermore, the health time of children can be expressed as a function of human capital health H, which is set as $h=\Omega -{\mathrm{BH}}_{t}^{-C}$, where B and C are constants satisfying $B\in \left(0,1\right)$ and $C\in \left(0,1\right)$. The health human capital H satisfies $H_{t+1}=I_{t}+\left(1-\delta_{t}\right)H_{t}$, where $\delta_{t}$ is the health human capital depreciation rate, $\delta_{t}\in \left(0,1\right)$. I represents the investment in health human capital, which is the output of medical service consumption M; namely, I=φM, where φ is the conversion rate, $\varphi \in \left(0,1\right)$. In this paper, the equilibrium conditions of child health investment are obtained by maximizing the child utility under budget constraints and time constraints:

(3) $$\frac{G_{t}\left(C_{c}/\Omega \right)}{\pi_{t-1}}+\frac{G_{t}\left(U_{\mathrm{ht}}/m\right)}{\pi_{t-1}}=\delta_{t}$$

where${\;G}_{t}=\partial {\mathrm{TL}}_{t}/\partial H_{t}$, which is the marginal product of health human capital; $U_{\mathrm{ht}}=\partial U/\partial H_{t}$; t is the marginal utility of health human capital; M is the marginal utility of money income; and $\pi_{t-1}$ is the shadow price of health.

**Figure 1:**
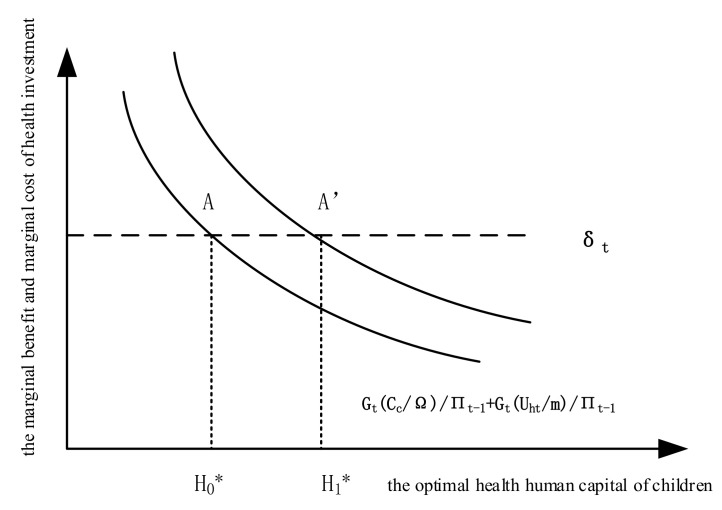
Effects of the New Rural Pension on children’s physical and mental health.

The two sides of Equation (3) can be understood as the marginal benefit and marginal cost of health investment, respectively, and the equilibrium point is the optimal health human capital of children; $H_{0}^{*}$, as shown at point A in Figure 1.

In summary, the elderly receiving the NRP will increase their care time T_c_ and transfer payments C_c_ to children, thus reducing the shadow price of child health investment, π_(t−1)_, and increasing the marginal returns to child health investment. This shifts the marginal return curve of child health investment to the right, and the equilibrium point shifts to the right with it (as shown at point A’ in Figure 1), at which time the health human capital of children increases from H_0_* to H_1_* (i.e., the health human capital of children is increased).

### 2.2. Social Pensions and Child Physical Health

Only a few studies have shown evidence indicating that the effect of social pensions on children’s physical health differs according to the level of economic development of the country [17,45,46]. In developed countries, such as the United States, social pensions, as a type of monetary transfer, have very little impact on child welfare [46]; alternatively, in developing countries, such as South Africa, social pensions can significantly improve the physical health of children in poor families, especially in terms of health outcomes including morbidity, height, and anemia [47]. One study has shown that the direct income transfers of poor households can contribute to an increase in human capital for children [17]. In addition to studies at the micro-level, a number of studies have shown that conditional cash transfers can improve the physical health interventions for citizens in low- and middle-income countries at the macro-level [48,49,50], which suggests that conditional monetary transfers are effective in improving access to and the use of physical health services, in addition to improving health outcomes in low- and middle-income countries. As a type of public monetary transfer, the NRP, which has achieved wide coverage in the rural areas of China, the largest developing country in the world, may also contribute to the physical health development of children. In view of the aforementioned research, we propose the following hypothesis regarding the effect of the NRP on children’s physical health in rural China.

**Hypothesis** **1.** *NRP can significantly improve the children’s physical health in rural China.*

### 2.3. Social Pensions and Child Mental Health

Studies have found the association between low family income and mental health problems in children [51,52,53,54,55,56,57,58,59,60]. Low family income may bring out persistent poverty that exerts a particularly strong cumulative and negative influence on the mental development of children. As a representative chronic family adversity, the lower the family income a child experiences, the more likely that difficulties in their peer relationships will appear, which badly affects their physical development and leads to low mental health levels [61]. Furthermore, lower family income may result in parental discord, parental psychosocial maladjustment, and a depressed family environment, which can also lead to accumulated harm to children’s mental health over a long period of time [62,63]. A child health survey also suggests that the proportion of children with mental health problems increases when family income decreases [64]. As a way to protect the low-income group, social pensions can provide extra income to the beneficiary’s family, especially in low-income countries and for elderly people [65,66]. We inferred that social pensions have a positive impact on children’s mental health development. We propose the following hypothesis regarding the effect of the NRP on children’s mental health in rural China.

**Hypothesis** **2.** *NRP can significantly improve the children’s mental health in rural China.*

### 2.4. Family Childcare Types and Child Health

Family childcare, as a vital part of the family environment, is one of the main factors in the development of children [67,68,69]. Studies have documented that early experiences with caregivers critically affect child development [68,70]. The various childcare types lead to different interactions between childcare and children [71,72,73]. Incremental involvement in caregiver–child interactions may be linked to an improved cognitive impact on the development of children in both physical and mental aspects, according to different types of family childcare [72,74,75,76]. In rural China, the children who are not taken care of by their parents all day can be divided into two groups: one group is taken care of by their grandparents full-time, thus having little chance to meet their parents (we call this type of childcare “full-time care” in this paper); the other group is taken care of by their grandparents part of the time, thus still having some time with their parents (we call this type of childcare “part-time care” in this paper). In the environment of intergenerational rearing, full-time and part-time care of children by the elderly have different amounts and patterns of interacting activities, which may lead to differences in the development conditions of children’s physical and mental health. Part-time care of children by the elderly can lead to more active interactions, by getting along with their parents, and the children can receive more emotional support from their mother, playful and physically stimulating interactions from their father, etc. [71,77,78]. In contrast, the full-time care of children by the elderly can lead to less chance of active interaction, as grandparents focus more on the diet, sleep, and other physiological demands of children, rather than mental demands [73,79]. In rural China, the two types of intergenerational family childcare (i.e., full-time and part-time care by elderly) both exist and have different effects on the development of children, such that the impact of the NRP on children’s health may have different outcomes under different family childcare types.

**Hypothesis** **3.** *NRP has a different impact on children’s physical and mental health between full-time childcare and part-time childcare.*

### 2.5. Child Gender Difference in Childcare

Several studies have shown that there are obvious differences between the healthy development levels of boys and girls in rural China [80], as the practice of preference for boys and discrimination against girls is still prevalent, especially among poor peasant families. Such beliefs about the superiority of boys have impacts on child-rearing practices, and boy preference values determine differential developmental opportunities for children inside families [81,82,83]. These beliefs about boys may result from traditional values (e.g., strong Confucian traditions), social customs (e.g., lineage ties and systems of dowry), and economic benefits (e.g., increased labor force opportunities, security of the families, and old-age support for parents), or from a combination of these various social, cultural, and economic attributes [84,85,86,87,88,89,90]. The complex interplay of economic and sociocultural factors determines the benefits and costs of a child. If the net utility of having a boy outweighs that of a girl, families are likely to prefer boys to girls [91], which is clearly reflected in food allocation, prevention of diseases and accidents, treatment of sick children, and other childcare actions [92,93,94,95]. Especially in poverty-stricken areas with limited resources, the preference for boys will cause families to allocate nutrition and healthcare preferentially to them [96]. At present, due to the unbalanced economic development in China, there are still resource limitations and relative poverty in rural China families, and the concept of childcare is still influenced by the beliefs of a preference for boys, especially among the elderly. Therefore, we propose that the impacts of the NRP on children’s physical and mental health vary with the gender of the child.

**Hypothesis** **4.** *NRP has different impacts on children’s physical and mental health that vary with children’s gender.*

## 3. The New Rural Pension Scheme in China

In September 2009, China State Council promulgated the “Guidance Opinions on Piloting New Rural Pension Scheme” and started piloting the New Rural Pension (NRP) Scheme. Under this scheme, rural residents who reach the age of 16 (excluding school students) and who do not register for the social pensions for urban workers can voluntarily join the NPR Scheme. By combining social pooling and individual accounts, the NRP (i.e., a funding model that encompasses individual contributions, collective subsidies, and government subsidies) was established. The pension consists of a basic pension and a personal account pension, which are paid for one’s whole life. The central government has set the basic pension standard at 55 yuan per person per month, and local governments can raise this basic pension standard, according to their social and economic level. For rural residents who have paid for a long time, the basic pension can be increased accordingly, which is covered by local governments. With the continuous expansion of the NRP pilot area, by the end of 2012, the NRP policy covered 480 million people in 2853 county-level administrative regions nationwide. Since then, the NRP and the urban pension scheme have gradually merged into one basic pension system. By the end of 2020, the number of participants in the basic pension system had reached 999 million nationwide.

As an important part of the social pension system of China, the NRP is designed and implemented by the government, with the aim to improve the welfare of the elderly in rural areas. Most of the existing studies have focused on the effects of the NRP on the elderly, such as their physical health, consumption, and subjective welfare [97,98,99,100]. For example, several studies have reported a positive causal relationship between the NRP and the physical health level of the elderly and its influencing mechanisms [101,102]. Other studies have found that the NRP increased the probability of the elderly receiving daily care and spiritual comfort [103], which further helped to reduce their depression [99].

However, research on the impact of the NRP on other family members, especially children, remains lacking [104,105]. Only a few studies have explored the impact of the NRP on families; for example, Chen et al. [106] and Zhang et al. [43] found that the NRP not only can enhance the welfare of the rural elderly, but also has a crowding-out effect on generational transfers and a substitution effect on family old-age care. Zheng et al. [34] suggested that the NRP has a significant impact on the physical health of rural children under the age of 15, with nutritional intake being the main influence path, which still needs more investigation. However, to date, the impact of the NRP on the mental health of rural children has been under-researched.

In the current context of intergenerational care in rural China, can the NRP received by older adults improve the physical and mental health of children? Are there gender differences in the impact of receiving the NRP on child health in rural China, where “boy preference” exists? What is the role of the time length of intergenerational care in the impact of the NRP on children? This study examines the impact of the NRP on the physical and mental health of rural children, and analyzes the heterogeneous impact of the NRP due to differences in the time length of intergenerational care and gender.

## 4. Methodology

### 4.1. Data

The data used in this study were obtained from the China Family Panel Studies (CFPS) in 2016. The survey was conducted by the China Social Science Survey Center (ISSS) of Peking University, covering 25 provinces (cities and districts), with a sample size of 14,019 households. This sample is representative of households throughout the whole country. The survey tracked and collected data at the individual, family, and county levels. Respondents included all family members in the sample households, and the questionnaire includes demographic characteristics of respondents and their information relating to migration, economic activities, family relations, family dynamics, etc. Therefore, these data can meet the needs of our study, regarding the impact of the NRP on the physical and mental health of rural children, because we need to know the receipt of the NRP by the elderly in each family, the physical and mental health of their grandchildren, and other basic information about the family.

The data were downloaded directly from CFPS official website, as it is a free, open-source database. As this paper focuses on the two generations of grandparents and grandchildren in rural families, we processed the data as follows: First, we selected the variables related to physical health, mental health, and basic information in the children’s database. Then, we matched the family ID, family basic information database, and family economic database in the children’s database to form a child–family database, in order to realize a one-to-one correspondence between the information of children and families. Next, we matched the personal IDs of children’s grandparents, according to the questionnaire of family basic information, and matched the information in the adult database according to the personal IDs of the grandparents, in order to realize a one-to-one correspondence between the information of children and grandparents, and to form a large sample set of children, families, and the elderly. Finally, after selecting and retaining rural families according to the locations of their home addresses, and deleting the samples with certain key variables, an effective sample of 2142 children was obtained.

### 4.2. Descriptive Statistics

The explained variables are children’s physical health and mental health levels. The physical health level was measured by the number of medical visits due to illness in the previous 12 months, and the mental health level was measured through the construction scores of five items of the Center for Epidemiologic Studies Depression (CES_D) scale. The explanatory variable was whether the elderly in this family received the NRP.

The descriptive statistics of the sample are reported in Table 1. The table shows that the average number of medical visits of children in the previous 12 months was 1.4; the average child CES_D scale score was 2.7; 37.9% of the respondents received the NRP; the average age of the elderly was 59 years old; and the gender ratio of children was roughly the same. The average age for children was 12.4 years old; nearly 15.0% of the children were taken care of by the elderly all day; and the average number of times the children talked with their parents was 1.8 times a month. The average level of health status of the elderly was in the middle, and the average education level of the elderly was low. The average score of the CES_D scale for the elderly was 34, and the average logarithm of per capita net income of the family was 8.9.

### 4.3. Model Setting

We used regression discontinuity (RD) to test the effect of the NRP on the physical and mental health of rural children. Among the econometric methods used to identify causal effects, regression discontinuity is a rigorous quasi-randomized trial method, which is often used in policy evaluation [107,108,109] and exhibits a number of advantages, such as bypassing many of the questions concerning model specification, assessing the possibility of manipulation of the assignment variable, and exploring the sensitivity of the results to the inclusion of baseline covariates [110,111,112]. The basic idea of this method is that the samples near both sides of the discontinuity are comparable in all characteristics. Making use of the discontinuous characteristics of policy rules, economic agents are treated when an observable characteristic variable (forcing variable) is equal to or greater than a certain threshold. As long as economic agents cannot fully manipulate the driving variables, a discontinuous change in the dependent variable can be considered to be caused by the processing state. According to “the Guiding Opinions on the Pilot Project of New Rural Pension Scheme” issued by the China State Council in 2009, the NRPS follows the age rule (i.e., the insured can only receive the NRP when they are over 60 years old), which means that the probability of the elderly receiving the NRP will increase significantly after the age of 60 years. This can be regarded as a “local random experiment” caused by the impact of exogenous policies, thus providing a favorable condition for the identification of causality and meeting the requirements of regression discontinuity design. Therefore, we used the following model:

(4) $$D_{i}=\left\{\begin{array}{c} 1\;\;z_{i}\geq 60\\ 0\;\;z_{i}<60 \end{array}\right.,$$

where *D_i_* is the processing status variable, which indicates whether the NRP is received. If it is equal to 1, it indicates receiving the NRP; otherwise, it is 0. Equation (4) shows that *D_i_* is a discontinuous function of the age *z_i_*, which is a discontinuity point; that is, no matter how close *z_i_* is to 60, *D_i_* will not change until *z_i_* equals 60. The variable *z_i_* refers to the driving variable, according to the literature [113]. If Equation (4) holds, the causal effect of the NRP on children’s health can be obtained by regressing the following equation:

(5) $$Y_{i}=\alpha +\rho D_{i}+f\left(z_{i}\right)+\varepsilon_{i},$$

where *f*(*z_i_*) is a polynomial function of *z_i_*. When Equation (4) holds, we call this Sharp RD. If the probability of an individual receiving treatment at the discontinuity point does not jump directly from 0 to 1, but jumps from a small probability *a* to another probability *B* (0 < *a* < *B* < 1), it is a fuzzy RD design. In the fuzzy RD design, whether the individual is treated does not depend entirely on whether the grouping variable is greater than the threshold. Some individuals below the threshold are treated, while some individuals above the threshold are not treated; that is, in many cases, although the processing state *D_i_* is a discontinuous function of the driving variable *z_i_*, it does not necessarily change from 0 to 1 at the discontinuity point, but only increases the probability that the value of *D_i_* changes. That is, *D_i_* and *z_i_* are related as follows:

(6) $$P\left[D_{i}=1|z_{i}\right]=\left\{\begin{array}{c} g_{1}\left(z_{i}\right)\;\;z_{i}\geq 60\\ g_{0}\left(z_{i}\right)\;\;z_{i}<60 \end{array}\right.\;,\;g_{1}\left(z_{i}\right)\neq g_{0}\left(z_{i}\right).$$

In the implementation of the NRP, there are also some individuals over 60 years old that do not receive the pension. Therefore, we assume that *g*_1_(*z_i_*) > *g*_0_(*z_i_*); that is, the probability of rural elderly people over 60 years old receiving the NRP is greater than that of those under 60 years old. In the case that Equation (6) holds, we call it fuzzy RD.

Fuzzy RD estimation can be achieved by two-stage least squares (2SLS), which is equivalent to instrumental variable (IV) estimation [114,115]. Specifically, the first-stage equation can be expressed as:

(7) $$PD_{i}=\delta +f\left(z_{i}\right)+\theta T_{i}+\mu_{i},$$

where *T_i_* = 1 (*z_i_* ≥ 60) and is the IV for the treatment state *D_i_*. The second-stage regression setting is the same as Equation (5). The reduced form equation can be obtained by substituting Equation (7) into Equation (6).

In practice, fuzzy RD estimation can be obtained by non-parametric IV estimation or parametric 2SLS estimation, and the two estimation methods are equivalent [110,113]. The parametric 2SLS estimation results are reported in this paper. When estimating, the requirement on the functional form of *f*(*z_i_*) can be relaxed by restricting the sample to the vicinity of the discontinuity point. The distance between the selected sample and the discontinuity point is called the bandwidth. The smaller the bandwidth, the smaller the requirements for the control variables and forms; however, more sample observations will be lost and the error of parameter estimation will be increased. In the subsequent estimation, according to Lei [116], segmented linear functions are used when controlling for age. The optimal bandwidth is calculated according to Imbens and Kalyanaraman [117], and three types of optimal bandwidth estimation results, according to the triangular kernel estimation, are reported to show the robustness of the results.

## 5. Empirical Results

### 5.1. Graphic Analysis

According to Zhang and Chen [43], we drew figures to show the relationships of driving variables with treatment state and outcome variables. The figures help to predict whether there is a jump between the treatment state and outcome variables at the age discontinuity point, and then to preliminarily speculate about the relationship between the NRP and children’s physical and mental health.

**Figure 2:**
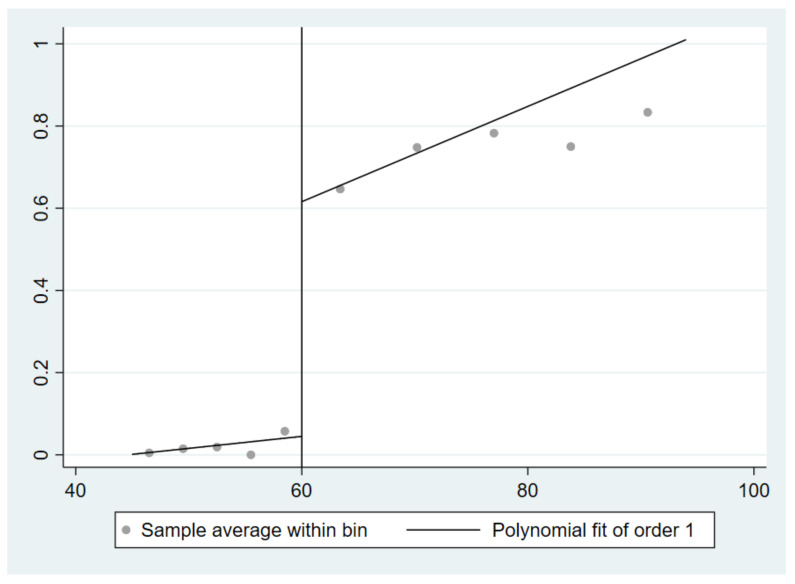
Relationship between receiving the NRP and the age of the elderly in rural areas.

Figure 2 shows the relationship between the age of the rural elderly and whether they received the NRP (i.e., the first stage of fuzzy RD estimation). We took 60 years old as the coordinate origin and found that there was an obvious jump in the proportion of those receiving the NRP near 60 years old. This result is consistent with the conditions stipulated in the NRPS.

**Figure 3:**
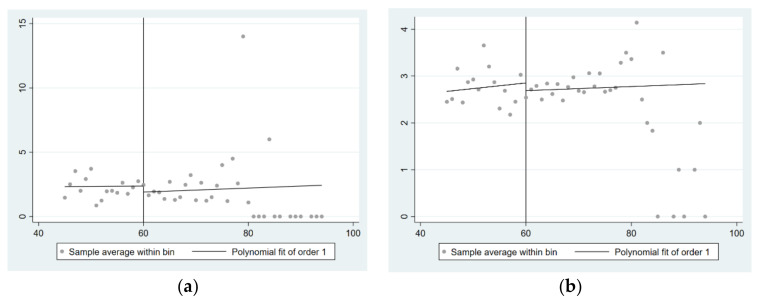
Relationship between the age of the elderly and children’s physical health and mental health: (**a**) The relationship between the number of children’s medical visits due to illness in the previous 12 months and the age of the elderly; and (**b**) the relationship between children’s CES_D scale scores and the age of the elderly.

Figure 3a shows the relationship between the number of medical visits of children due to illness in the previous 12 months and the age of the elderly, and Figure 3b shows the relationship between children’s CES_D scores and the age of the elderly. We can see obvious downward jumps in Figure 3a,b, indicating that children’s physical and mental health may be improved at the age discontinuity point. Nonetheless, regression analysis is still needed, in order to verify whether there exists a significant relationship between the NRP and the physical and mental health of rural children.

### 5.2. Regression Discontinuity of Overall

The results of the graphical analysis indicated that the NRP may have a negative impact on the number of children’s medical visits in the previous 12 months and their CES_D scores. Therefore, discontinuity regression was further conducted to verify this result.

Table 2 reports the relationship between the NRP and the number of children’s medical visits in the previous 12 months. The first line reports the impact of the age rules of the NRPS on whether the rural elderly received the NRP. The estimation result under the optimal bandwidth demonstrates that, when the age of the rural elderly meets the conditions for receiving the NRP (i.e., 60 years old), their probability of receiving the NRP will be significantly increased (by 41.53%). The estimation results under the 3/4 optimal bandwidth and the 5/4 optimal bandwidth were basically the same. The second row reports the impact of the age of the elderly on the number of children’s medical visits in the previous 12 months. The estimation result under the optimal bandwidth shows that the number of children’s medical visits in the previous 12 months near the discontinuity point significantly decreased (by 281.83%). Similar results were reported for estimates under the 3/4 optimal bandwidth and the 5/4 optimal bandwidth. These results show that the NRP can significantly improve the physical health of children in rural China. Therefore, Hypothesis 1 was supported.

Table 3 reports the relationship between the NRP and the children’s CES_D scale scores. The first line reports the impact of the age rules of the NRPS on whether the rural elderly received the NRP. The estimation result under the optimal bandwidth shows that when the age of the rural elderly met the conditions for receiving the NRP (i.e., 60 years old), their probability of receiving the NRP was significantly increased (by 44.72%). The estimation results under the 3/4 optimal bandwidth and the 5/4 optimal bandwidth were basically the same. The second row reports the effect of age on children’s CES_D scale scores. The estimation result under the optimal bandwidth shows that children’s CES_D scale scores near the discontinuity point decreased significantly (by 221.38%). Similar results were reported for estimates under the 3/4 optimal bandwidth and the 5/4 optimal bandwidth. These results show that the NRP can significantly improve the mental health of children in rural China. Therefore, Hypothesis 2 was supported.

In summary, the regression discontinuity results showed that the NRP had a significant and negative impact on the number of children’s medical visits in the previous 12 months, in addition to a significant and negative impact on children’s CES_D scale scores, indicating that the NRP improved both their physical and mental health. Thus, the rural elderly receiving the NRP can improve both the physical and mental health of rural children.

### 5.3. Effectiveness Test

#### 5.3.1. Continuity of the Driving Variable

**Figure 4:**
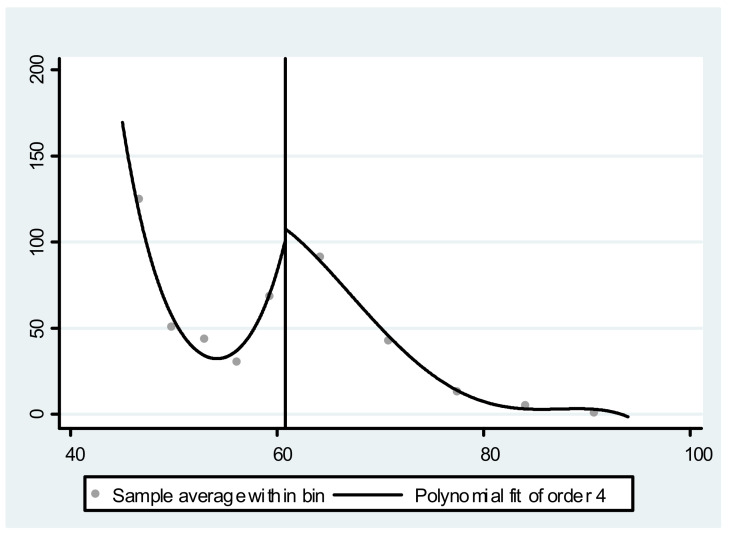
Age probability density curve.

The effectiveness of RD identification strategies requires that economic agents cannot manipulate (or, at least, cannot fully manipulate) the driving variables. In this study, if age can be manipulated (e.g., the elderly can lie about their age in order to get the NRP in advance), then the RD estimation is likely to be biased. One way to test whether their age is manipulated is to draw a figure to show the age density. If the elderly lied about their age, there will be an obvious discontinuity point in the neighborhood of 60 years old, as reflected in the figure. Figure 4 shows that, in this study, the age density function had no obvious jump in the neighborhood of 60 years old, indicating that there was no manipulation of the driving variable.

#### 5.3.2. Continuity of the Predetermined Control Variables

Another way to test the effectiveness of the RD identification strategy is to test the continuity of control variables. Although we concluded that the NRP has no significant effect on intergenerational care, it is still necessary to test this result. On one hand, testing the continuity of control variables is a vital step to show the effectiveness of RD identification strategy. On the other hand, it is likely that a control variable also jumps at the age discontinuity point in the opposite direction, and its impact on intergenerational care is opposite to that of the NRP on intergenerational care, which makes the RD results non-significant, after neutralizing the two opposite effects. Generally, control variables such as the health status of the elderly, the highest education level of the elderly, per capita net household income, child gender, child age, the number of times children communicated with their parents in the previous month, the CES_D score of the elderly, and whether children were taken care of by the elderly all day, may affect the physical and mental health of children, but there should be no jump at the age discontinuity point.

Table 4 reports the results of the test for continuity of the control variables. The RD setting was the same as above, and the explanatory variable was replaced by the control variables. The results show that there was no significant jump in all variables at the age of 60, except that the health status of the elderly was 10% significant at the 3/4 and 5/4 bandwidths. According to the actual situation, the health level of the elderly will not suddenly decline at the age of 60, so we infer that this situation is an accidental result of data analysis. If this inference is true, the continuity tests for the prior control variables all passed.

#### 5.3.3. Other Ages as Discontinuity

We further changed the location of the elderly’s age discontinuity point and set the discontinuity point to 55 or 65 years old. Through counterfactual analysis, we re-estimated the explained variable with fuzzy RD. That is, in order to verify the results of the above RD analysis, in an age change beyond 60 years old, there should be no obvious jump in the number of children’s medical visits in the previous 12 months and their CES_D scale scores.

Table 5 reflects the results of RD estimation with 55 and 65 years old as the discontinuity points. The local average treatment effects of the two explanatory variables (i.e., the number of children seeking medical treatment in the previous 12 months and the children’s CES_D scale scores) were not significant, indicating that there was no jump in the explanatory variables in the age that did not meet the requirements of receiving the NRP. Therefore, the placebo test also passed.

### 5.4. Heterogeneity Analysis

Investigating the heterogeneous associations between the NRP and children’s health is beneficial to better understand the effects of the NRP policy in rural China [34]. Thus, the impact of the NRP on children’s physical and mental health was further heterogeneously analyzed, according to the types of family childcare and the gender of children.

#### 5.4.1. Heterogeneity Analysis of Family Childcare Types

First, according to the different types of intergenerational family childcare (i.e., full-time and part-time care by the elderly), the total sample was divided into two sub-samples for fuzzy RD analysis, and the results are shown in Table 6. There was a distinct difference between full-time care and part-time care, in terms of the number of children’s medical treatments in the past 12 months when the elderly received the NRP. Specifically, when children were cared for by the elderly all day, the effect of receiving the NRP by the elderly on the number of children’s medical treatments was not significant. In contrast, when children were not cared for by the elderly all day, the elderly receiving the NRP had a significant impact on the number of children’s medical treatments.

Similarly, the impact of the NRP on children’s mental health under different intergenerational childcare types is detailed in Table 7. In both full-time and part-time care sub-samples, there was a significant and negative correlation between the elderly receiving the NRP and the children’s CES_D scale scores; that is, regardless of whether the children were cared for by the elderly all day, the NRP significantly improved the mental health of rural children in both sub-samples. However, the coefficients for the two sub-samples were different. Under the same optimal bandwidth, the absolute value of the coefficient for the effect of the NRP on the CES_D scale score for children cared for by the elderly all day (−6.1265) was much higher than that for children not cared for by the elderly all day (−0.7383), with a difference of 7 times. This suggests that, in terms of children’s mental health, the impact of the NRP on children who are cared for by the elderly all day is much greater than that on children who are not cared for by the elderly all day.

In summary, the impact of the NRP on rural children’s health varies with different types of family childcare. When children are cared for by the elderly full-time, the NRP only improved their mental health significantly; alternatively, for the children who are cared for by the elderly part-time, the NRP not only raises their mental health level, but also improves their physical health level. These findings indicate that the NRP has different impacts on children’s physical and mental health, with respect to different family childcare types. Therefore, Hypothesis 3 was supported.

#### 5.4.2. Heterogeneity Analysis Regarding Gender of the Children

The improvement in children’s physical and mental health due to their grandparents receiving the NRP may also differ among children of different genders. The total sample was re-divided into two sub-samples of boys and girls, in order to estimate the marginal effect of the elderly receiving the NRP on reducing the number of illnesses of their grandchildren. The results regarding physical health are shown in Table 8. For boys, the effect of whether the elderly received the NRP on their physical health was not significant, although the marginal effect was positive. This indicates that the elderly receiving the NRP does not significantly reduce the number of medical treatments for their grandsons. In contrast, for girls, the effect of whether the elderly received the NRP on their physical health was significant at the level of 1%. This indicates that the elderly receiving the NRP significantly reduces the frequency of medical treatments for their granddaughters.

We also estimated the marginal effect of the elderly receiving the NRP on reducing the CES_D scale scores of their grandchildren, for which the results are shown in Table 9. Although the coefficients were negative for both boys and girls, indicating that the level of mental health was improved in both groups, the effect of the NRP on the CES_D scale scores was only significant for girls, under the optimal bandwidth and 3/4 optimal bandwidth. Therefore, the impact of the NRP on children’s mental health was only significant for the group of girls. This indicates that, when the elderly receive the NRP, they only made effective efforts to improve the mental health levels of their granddaughters, but not their grandsons.

In summary, the effects of the NRP on the health of rural children vary with the gender of the child. For the girl group, when their grandparents received the NRP, their physical and mental health levels were both improved significantly and sufficiently; however, for the boy group, only physical health was significantly impacted by the NRP, whereas their mental health level was not strongly affected. These results indicate that the NRP has varying impacts on children’s physical and mental health, which vary with child gender. Therefore, Hypothesis 4 was supported.

## 6. Discussion

This study contributes to the existing literature on the impact of social pensions on the health of children by analyzing the intergenerational effects of the NRP in the context of rural China. In terms of child health, we focused on both the physical and mental health of rural children, thus filling a research gap on the effects of the NRP, given that studies on the impact of the NRP on children’s physical and mental health are limited. Furthermore, we not only examined the effect of the NRP on the physical and mental health of children, but also theoretically analyzed the possible effect mechanism and tested the heterogeneity. Moreover, we adopted the fuzzy RD strategy to address the possible endogeneity problem.

First, we verified the effect of the NRP on the physical health of children. We found that receipt of the NRP in rural families had positive and significant impacts on the physical health level of children, in that it can greatly decrease the number of medical visits. This echoed other studies regarding the value and impact of social pensions on child health in China [34] and in other countries [17,18,45], demonstrating the vital role that social pensions play in the physical health of family members other than the receiver. The reason for this is that, when the rural elderly receive the NRP—which can be regarded as an additional source of family income [65]—their labor burden is reduced and their daily leisure time is increased [43]. Therefore, the rural elderly can increase their childcare time and pay more attention to the daily demands of children, which can improve their living conditions and facilitate their healthy growth (e.g., having more time to take children out for outdoor sports, which can improve their physical fitness and reduce their chance of becoming sick) [73,79]. Furthermore, the NRP can also directly increase the disposable income of families, to a certain extent [30,31], which can increase investments in child health (i.e., improving the family’s medical resources and level, such that the children have better preventive medical and healthcare conditions) [17]. Therefore, greater attention needs to be paid to the effect of social pension systems on the physical health of children in the rural China context.

Second, in this study, we also verified the effect of the NRP on the mental health of children. We proved that receipt of the NRP in rural families also had a positive and significant impact on children’s mental health levels, as it can greatly decrease the CES_D scores of children (the lower the CES_D scores, the higher the level of mental health). These results are in concert with studies that mentioned the impact of family income and transfer payments on children’s mental health [61], and proved the association between low family income and children’s mental health problems [51,52]. When the elderly receive the NRP—which means that their family income increases—their working hours can be reduced (i.e., working in the fields). Therefore, they have more time and energy to accompany and care for their grandchildren, such as having more opportunities to actively interact with their grandchildren, which may enhance the children’s psychological security and happiness, thus contributing to their mental health [36,117] and increasing their subjective welfare [117,118]. In addition to the increase in childcare time, the NRP also directly increases the disposable income of families [30,31], such that the elderly are more likely to meet children’s small material needs (i.e., buy them little gifts), which can provide children with more material satisfaction [119], thus resulting in more peer social satisfaction and psychological security. Therefore, we suggest that the effects of social pension systems on the mental health of children also need to be paid more attention in the rural China context.

Third, we found that the childcare type has an influence on the impact of the NRP on the physical and mental health of children in rural China. Regarding physical health, when children were under part-time childcare (i.e., elderly do not take care of the children all day), their physical health level was significantly improved when the elderly received the NRP; however, this result was not observed for the full-time childcare group, in contrast to the findings of Yu [35]. This can be explained according to two aspects: First, the elderly’s care of children may be unscientific, and there is a lack of opportunities for the children’s parents to correct this when the children are cared for by the elderly all day. Therefore, it may not be beneficial to the children’s physical health. In contrast, when children are cared for by the elderly on a part-time basis, they will be cared for alternately by the elderly and their parents. In this way, unscientific care by the elderly is likely to be corrected by the children’s parents, which can contribute to the physical health of children, providing evidence for the existing theoretical research [67,68,69]. Regarding mental health, the impacts of the NRP on the mental health of rural children were both significant and positive in the part-time and full-time childcare groups. However, compared to children under part-time care, the initial health level of children under full-time care was lower [120]. Therefore, as the initial physical condition of the children was not good, the improvement in their mental health level is probably more obvious. We speculate that the reason for this result is that when the elderly receive the NRP, they can spend more time on childcare and have more money to provide spiritual satisfaction and pleasure to their grandchildren, such as giving them pocket money or buying them little gifts. Compared to children who are under part-time childcare, the children under full-time childcare would have more time to spend with their grandparents and, thus, they have more chance to access the benefits of the NRP. As one of the main forces in family childcare, grandparents are often sidelined in research and policy initiatives, despite the primary or secondary caregiving roles they often assume. Thus, this study adds to our understanding of the effects of different types of childcare on the physical health of children in rural China [121,122], and provides evidence regarding the varying effects of the NRP on the mental health of rural children, thus filling the gap in this direction.

Finally, in this study, we also showed that the impact of the NRP varies with the gender of children in rural China. There were significant impacts on the physical health of both girls and boys, while the impact on mental health was only significant for girls. Regarding physical health, the improvement in the physical health level in girls was significantly higher than that in boys. One possible reason for this is that, because “boy preference” exists in rural areas, when the care time of the elderly is limited, this time is more preferentially given to boys, thus reducing the time for girls. In this case, the health status of girls is significantly lower than that for boys [123]. However, when the elderly receives the NRP, their total care time for their grandchildren increases, and the gender discrimination in the allocation of care time can be alleviated. Therefore, the physical health of girls may be improved. Regarding mental health, the impact on NRP was only significant for girls, which may also be related to the traditional concept of “boy preference” in rural China. Specifically, the elderly pay more attention to the development of boys, such that the boys have a higher level of initial mental health due to higher care, and the girls have a lower level of mental health due to having received less attention. The NRP gives the elderly more leisure time through the substitution effect and income effect. As a result, they not only can take care of their grandsons, but also increase their attention to and financial support for their granddaughters. Thus, the NRP can significantly improve the mental health of girls. In addition, girls are more psychologically sensitive, such that the feedback from caregivers is more obvious [124], which can also serve as an explanation. Overall, the gender of the child resulted in different outcomes regarding the effect of the NRP on the health of rural children, on the basis that both groups had positive effects, whereas the impact on mental health was more effective for girls. Thus, welfare policy-makers should fully consider the gender balance of the beneficiary children under the realistic cultural background and limited economic conditions. Family childcare givers should also be balanced in terms of taking care of children, and equality between girls and boys should be achieved in family resource allocation and emotional value output.

## 7. Conclusions

In this study, we evaluated the influence of the NRP policy on the development of children in rural families under the context of universal intergenerational support and childcare in rural China, and the mechanisms driving its outcomes. The results indicated that the receipt of the NRP by the elderly had a significant and positive effect on both the physical and mental health of rural children. The effect of the NRP on the health level of children differed according to the family childcare types; that is, in the part-time childcare group, reception of the NRP by the elderly positively affected both the physical and mental health of children whereas, in the full-time childcare group, the NRP only had a significant and positive impact on their mental health. The effect of the NRP on the health level of children also differed according to the gender of children; that is, for the boys, only their physical health was significantly and positively impacted when their grandparents received the NRP whereas, for girls, the NRP significantly improved both their physical and mental health levels. The results of this study serve to enhance our understanding of the effects of the NRP on children, in addition to emphasizing the importance and urgency of paying attention to the health of rural children in the context of intergenerational childcare. Moreover, it should be noted that the family childcare types and children’s gender play an important role in children’s physical and mental health development.

Our study offers several implications for enhancing the health of rural children through the development of the social pension system. First, the significant role of social pensions in improving the physical and mental health of rural children should be emphasized. Because we found that the NRP positively affected both the physical and mental health of children, the establishment of a social pension system may be used as an effective approach to enhance the development of rural children [34,118,125]. As such, governments should make more efforts to improve the social pension system in their own context [126,127,128,129]. In addition, social insurance programs for rural children could also be established to improve their health. Second, the effects of family childcare type in conjunction with social pensions on child health should be recognized. Children should be differentiated when using social pensions to enhance their health. For children under full-time childcare, a more scientific manner of care should be provided [121,130,131,132,133], considering that the unscientific care of the elderly may account for the non-significant effect of the NRP on their physical health. For children under part-time childcare by the elderly, given that the impact of the NRP on their mental health was significantly lower than that of children under full-time care, as shown in this study, increasing pensions may be a useful way to enhance children’s mental health, as it can help the elderly to spend more time and money on accompanying and raising them. The study also reminds policy makers to popularize scientific family childcare knowledge and methods in rural China, and provide a better environment for rural children’s growth by enhancing the childcare knowledge of their parents and grandparents. Third, the “boy preference” phenomenon in rural China was found to affect the impact of the NRP on the healthy development of children, both physically and mentally. Because this study revealed that the effect of the NRP on the physical and mental health of girls was significantly higher than that for boy, increasing pensions may also be effective for further enhancing the physical and mental health of girls. Furthermore, more attention needs to be paid to the welfare of girls and the uneven development of boys and girls in rural China. This may also serve to provide ideas for policies focused on children’s development. Finally, because our theoretical inferences showed that the elderly who receive the NRP can improve their grandchildren’s physical and mental health by increasing the time spent on childcare and transfer payments, the social welfare of the elderly—more than their pensions—should be further improved, in order to ensure their leisure time and disposable income [134,135,136,137]. The welfare of vulnerable children’s other caregivers also needs to be improved to enhance the development of children in underdeveloped areas.

Readers should be reminded of the limitations of this study. First, due to the limitations of the database, there was a lack of data from areas where the NRP has not been implemented, such that falsification tests could not be conducted by using these areas as a control. Second, because we used cross-sectional data and the results were limited to the effects of the NRP on children’s physical and mental health over only one year, research on the long-term effects of the NRP should be conducted in the future. Third, given the complexity of the effects of the NRP within households and the possible lags in such effects, a more in-depth investigation of the effects of the NRP on children’s health from a household perspective is needed; for example, studies of the effect of the specific amount and proportion of the NRP spent on the elderly on children’s health, the different influencing mechanisms and priorities of NRP to improve children’s health levels, the influence of NRP on other aspects of children (e.g., academic performance, cognitive ability, etc.), and the specific mechanism of the impact of NRP on children. Fourth, although we used a list of factors that may influence the children’s physical and mental health as control variables, other factors, such as the children’s school education, may also affect their physical and mental health, which should be further explored in future studies. Finally, although the study tried to eliminate the assumed errors, i.e., the use of RD to better deal with the problem of endogeneity, the robust results under different bandwidths, and both the prior and comfort tests, future research using other data sources and methods (e.g., PSM-DID) should be conducted to verify the findings of this study.

## Figures and Tables

**Table 1:** Descriptive statistics of the sample.

Variable	Obs	Mean	Std. Dev.	Min	Max
Medical visits of children in the previous 12 months	2142	1.3744	2.5296	0.0000	30.0000
Child CES_D scale score	2142	2.7269	2.1792	0.0000	12.0000
Elderly receive the NRP	2142	0.3786	0.4852	0.0000	1.0000
Elderly age	2142	59.0724	9.9170	45.0000	94.0000
Child gender	2142	0.5369	0.4988	0.0000	1.0000
Child age	2142	12.3543	1.6940	10.0000	15.0000
If taken care of by the elderly all day	2142	0.1629	0.3694	0.0000	1.0000
Times of children talking with their parents	2142	1.8240	3.9693	0.0000	30.0000
Elderly health status	2142	3.3973	1.2637	1.0000	5.0000
Elderly education level	2142	1.7316	0.9302	1.0000	5.0000
Elderly CES_D scale score of children	2142	33.9136	8.5792	20.0000	72.0000
Logarithm of per capita net income of the family	2142	8.9235	0.9023	3.3440	14.5148

**Table 2:** Effect of the NRP on the number of children’s medical visits in the previous 12 months.

Variable	Optimal Bandwidth	3/4 Optimal Bandwidth	5/4 Optimal Bandwidth
Medical visits of children in the previous 12 months			
The impact of elderly aged ≥ 60 on receiving NRP	0.4153 ***	0.3620 ***	0.4583 ***
	(0.0344)	(0.0364)	(0.0324)
The impact of receiving NRP on medical visits of children in the previous 12 months	−2.8183 *	−3.7630 *	−2.0711 *
	(1.5113)	(2.0411)	(1.1925)
Observation	543	413	663

Note: The standard error of aggregation robustness calculated at the provincial level is in parentheses. *** *p* < 0.01, * *p* < 0.1. All regressions were controlled for child gender, child age, whether the child had commercial health insurance, residence, whether the child was cared for by the elderly on a full-time basis, the number of times the child talked with his parents in the past month, the health status of the elderly, the highest educational level of the elderly, the marital status of the older adults, the CES_D scale score of the older adults, and the per capita net household income (log). The optimal bandwidth for the first column was calculated using the default triangular kernel of the Stata software.

**Table 3:** Effect of the NRP on children’s CES_D scale scores.

Variable	Optimal Bandwidth	3/4 Optimal Bandwidth	5/4 Optimal Bandwidth
child CES_D scale score			
The impact of elderly age ≥ 60 on receiving the NRP	0.4472 ***	0.38780 ***	0.4921 ***
	(0.0331)	(0.0359)	(0.0304)
The impact of receiving the NRP on child CES_D scale scores	−2.2138 *	−3.0888 *	−1.5494 *
	(1.0986)	(1.6326)	(0.8357)
Observation	663	543	778

Note: The standard error of aggregation robustness calculated at the provincial level is in parentheses. *** *p* < 0.01, * *p* < 0.1.

**Table 4:** Hypothesis testing: control variables’ continuity testing.

Variable	Optimal Bandwidth	3/4 Optimal Bandwidth	5/4 Optimal Bandwidth
Elderly health status	−0.4284	−1.0609 *	−0.1239 *
	(0.5323)	(0.5983)	(0.4914)
Elderly education level	0.1911	0.1636	0.1100
	(0.3923)	(0.5237)	(0.3216)
Logarithm of per capita net income of the family	1.1913	1.5470	0.7458
	(0.8384)	(1.0955)	(0.5450)
Child gender	−0.2054	−0.0827	−0.1555
	(0.6811)	(0.8233)	(0.5968)
Child age	−0.4572	−0.4484	−0.2708
	(0.2838)	(0.3761)	(0.2243)
Elderly CES_D scale score of children	−0.6519	−0.5296	−0.2414
	(2.6967)	(3.2208)	(2.3762)
Times of children talking with their parents in the last month	−1.7703	−2.9444	−1.5650
	(2.3163)	(3.4114)	(1.7340)
Child taken care of by the elderly all day	−0.2259	−0.2457	−0.1517
	(0.2514)	(0.3269)	(0.1783)

Note: The standard error of aggregation robustness calculated at the provincial level is in parentheses. * *p* < 0.1. The coefficients at the age discontinuity point (age ≥ 60 years) are reported in the table, and the first column on the left is the explanatory variable of the regression. Sample selection and selection of the optimal bandwidth for each column are the same as those in Table 2.

**Table 5:** Placebo test: changing the age discontinuity point.

Discontinuity Points		55 Years Old			65 Years Old		
Dependent Variable	Statistic	Optimal Bandwidth	3/4 Optimal Bandwidth	5/4 Optimal Bandwidth	Optimal Bandwidth	3/4 Optimal Bandwidth	5/4 Optimal Bandwidth
The impact of receiving NRP on medical visits of children in the previous 12 months	Coef.	−7.2516	−6.2857	−12.3616	−1.0656	−1.5241	−6.0699
	Std. Dev.	27.2137	42.2935	18.3992	7.2060	5.5335	39.8750
	observation	375	277	513	755	612	876
The impact of receiving NRP on child CES_D scale score	Coef.	−19.2840	−97.4072	−16.8571	2.0167	3.1471	5.0414
	Std. Dev.	23.1454	820.7924	12.5158	5.9477	4.8753	17.0556
	observation	375	277	513	755	612	876

**Table 6:** Impact of receiving the NRS on the number of medical visits by children in the previous 12 months, in terms of whether the elderly take care of the children all day.

Variable	Optimal Bandwidth	3/4 Optimal Bandwidth	5/4 Optimal Bandwidth
A: Full-time care			
The impact of elderly age ≥ 60 on receiving the NRP	0.4879 ***	0.4297 ***	0.5214 ***
	(0.0618)	(0.0689)	(0.0564)
The impact of receiving the NRP on medical visits of children in the previous 12 months	0.3289	−0.0082	0.6285
	(1.8573)	(2.6143)	(1.5508)
Observation	177	145	200
B: Part-time care			
The impact of elderly age ≥ 60 on receiving the NRP	0.4146 ***	0.3593 ***	0.4570 ***
	(0.0407)	(0.0437)	(0.0383)
The impact of receiving the NRP on medical visits of children in the previous 12 months	−4.0780 **	−5.0623 **	−3.2564 **
	(1.8601)	(2.4581)	(1.4872)
Observation	398	303	486

Note: The standard error of aggregation robustness calculated at the provincial level is in parentheses. *** *p* < 0.01, ** *p* < 0.05.

**Table 7:** Effect of the NRP on children’s CES_D scale scores, with respect to whether the elderly take care of the children all day.

Variable	Optimal Bandwidth	3/4 Optimal Bandwidth	5/4 Optimal Bandwidth
A: Full-time care			
The impact of elderly age ≥ 60 on receiving the NRP	0.4879 ***	0.4297 ***	0.5214 ***
	(0.0618)	(0.0689)	(0.0564)
The impact of receiving the NRP on children’s CES_D scores	−6.1265 **	−10.1849 **	−4.0853 *
	(2.9882)	(4.7650)	(2.1744)
Observation	145	110	177
B: Part-time care			
The impact of elderly age ≥ 60 on receiving the NRP	0.4146 ***	0.3593 ***	0.4570 ***
	(0.0407)	(0.0437)	(0.0383)
The impact of receiving the NRP on children’s CES_D scores	−0.7383 **	−0.8525 **	−0.6048 **
	(0.8786)	(1.2282)	(0.7063)
Observation	486	398	578

Note: The standard error of aggregation robustness calculated at the provincial level is in parentheses. *** *p* < 0.01, ** *p* < 0.05, * *p* < 0.1.

**Table 8:** The impact of the NRP on the number of medical visits of children in the previous 12 months, with respect to the gender of the child.

Variable	Optimal Bandwidth	3/4 Optimal Bandwidth	5/4 Optimal Bandwidth
A: Girls			
The impact of elderly age ≥ 60 on receiving the NRP	0.4207 ***	0.3755 ***	0.4629 ***
	−0.052	−0.0557	−0.0484
The impact of receiving the NRP on medical visits of children in the previous 12 months	−4.9012 **	−5.8674 *	−4.0849 **
	−2.5027	−3.1231	−2.0259
Observation	259	195	259
B: Boys			
The impact of elderly age ≥ 60 on receiving the NRP	0.4778 ***	0.4213 ***	0.5113 ***
	−0.042	−0.0458	−0.0387
The impact of receiving the NRP on medical visits of children in the previous 12 months	−0.5388	−0.9909	−0.2175
	−1.2151	−1.6578	−1.0248
Observation	344	284	453

Note: The standard error of aggregation robustness calculated at the provincial level is in parentheses. *** *p* < 0.01, ** *p* < 0.05, * *p* < 0.1.

**Table 9:** The impact of the NRP on the CES_D scale scores of children, with respect to the gender of the child.

Variable	Optimal Bandwidth	3/4 Optimal Bandwidth	5/4 Optimal Bandwidth
A: Girls			
The impact of elderly age ≥ 60 on receiving the NRP	0.4207 ***	0.3755 ***	0.4629 ***
	(0.0520)	(0.0557)	(0.0484)
The impact of receiving the NRP on children’s CES_D scores	−2.3034 *	−3.4664 *	−1.5498
	(1.2670)	(1.8952)	(0.9761)
Observation	319	259	375
B: Boys			
The impact of elderly age ≥ 60 on receiving the NRP	0.4778 ***	0.4213 ***	0.5113 ***
	(0.0420)	(0.0458)	(0.0387)
The impact of receiving the NRP on children’s CES_D scores	−2.3511	−3.7710	−1.6464
	(1.5864)	(2.4005)	(1.1958)
Observation	344	284	403

Note: The standard error of aggregation robustness calculated at the provincial level is in parentheses. *** *p* < 0.01, * *p* < 0.1.

## Data Availability

This study uses 2016 China Family Panel Studies (CFPS) data, “China Family Panel Studies” at https://www.isss.pku.edu.cn/cfps/ (25 March 2022).
